# A Case of Death from Chloroform

**Published:** 1853-12

**Authors:** T. K. De Wolf

**Affiliations:** Chester, Mass.


### BUFFALO MEDICAL JOURNAL

AND

### MONTHLY REVIEW.

VOL. 9. DECEMBER, 1853. NO. 7.

### ORIGINAL COMMUNICATIONS.

ART. I.—*A Case of Death from Chloroform.* Reported by T. K. De Wolf, M. D., Chester, Mass.

I have no disposition to startle the public mind by my caption, or the following facts; but it seems to me very proper, that every fatal case from chloroform should be carefully registered, that those in the habit of using it freely, not to say carelessly, may see to it, that an agent so potent in disturbing the healthy functions is never trifled with.

I was called into an adjoining town in consultation with my friends, Doctors Freeland and Smith. The patient was a young lady of about 25 years, of full and vigorous health, and in her second accouchment. I found her dying, but conscious, and obtained from her physicians the following history:

Some thirty hours before, doctor Freeland was called in and found her in the "preparatory" stages of active labor.

For several hours there was very little development of the case, and the patient became importunate for chloroform, having inhaled it during her first parturition. The doctor explained her present condition, and advised her that *now* was an improper time for the use of it, and after awaiting few hours, bled her from fifteen to twenty ounces. At this period, the case seemed to have made but little progress, and after an anodyne of some forty drops of tr. opii, she obtained some rest.

When she awoke she complained of pain in the abdomen and loins, and again importuned for chloroform. Strong and full pulse, not exceeding 100; tongue moist and clean; uterine action rather tardy; os uteri yielding; head advanced; pelvis roomy, and no unpleasant symptom. Under these circumstances the doctor promised her speedy relief, and persuaded her to take a decoction of the ergot. Very soon she insisted on having the chloroform, and sent a messenger for Dr. Smith. The doctor came, and brought as requested, a small bottle of chloroform, containing, as he believes, not more than ℥ij. He put it upon a table in sight of the patient, and while listening to doctor Freeland's narrative of facts in the case, the patient instructed a female friend to give her the bottle, and refused to give it back.

She inhaled, from time to time, and when told by both physicians that by persisting in the use of it, she would peril the successful termination of her labor, and possibly her life, her reply was: "My pains are quite comfortable." And in this condition remained about twelve hours.

Upon a careful examination, no material change in arterial action or nervous power was discovered, but very clearly, as they thought, a promising change in the rigidity of organs, and the chloroform being gone, they felt confident there would soon be increased uterine action, and a triumphant finishing up of the case. Alas! they were soon to be released, and their patient too. Now it was, that absence of all pain, a cold sweat, cold extremities, oppressed and whizzing respiration, receding pulse and "vacant glare," pointed to a sudden and fatal termination. All their friction, hot appliances, and active stimulants, were of no avail. I looked upon the dying woman with feelings of deep sorrow, for in her history I could see nothing, aside from the chloroform, to bring before me such an end, and hence, I came to the following conclusions:

1st. The *time* of her suffering would not have done it.

2d. The *amount* of her suffering would not have done it.

3d. There had been no rash quackish meddling.

4th. There was no rupture of vagina or uterus.

5th. There was no evidence of cerebral congestion from plethora, or other cause.

6th. Patient perfectly conscious, but insensible to pain; and

Finally. Her death, as it seemed to me, could be chargeable to nothing but the abolition of vital force, from frequent repetition of partial anæsthesia.

I have said she was perfectly conscious, and here is the evidence: She knew they had sent for me, and on my arrival I met the physicians in an adjoining room, and while listening to the facts above written, there came in a lady and said the patient desired to see me. In surprise, I asked *how is this?* The answer was, *she is positively dying, but conscious.* As I came into her presence she anxiously inquired, "O doctor! can you take my child and save me?" I very soon assured her I could take the child, and did so. To take the child, was *then* quite easy—but to *save her*, was impossible. The child, a fine boy, was dead, and in ten minutes the anxious mother was a corpse.

I am the more disposed to give you this case, from reading quite recently "Thoughts on Chloroform," by Prof. Gilman, of New York, (Buffalo Med. Jour., vol. viii., page 376.) In speaking of the use of chloroform, he says: "Never in natural, and very rarely in obstetric operations, do I go beyond a decided benumbing of sensibility." This was precisely the point arrived at by this woman, and during the entire period of the using, never for a moment lost her consciousness. The learned Prof. goes on: "This may or may not be attended with loss of consciousness." And again: "I have seen consciousness perfect, and sensibility entirely gone, and *vice versa. The rule then should be*, go always beyond the stage of excitement, till this is passed, nothing can be done, but as soon as it is passed, pause, and omit the progress, always short of stertor. Such I believe to be the true practice in obstetrics, and under it *no fatal case has occurred.*"

Now sir, be it remembered, I disclaim any intention of finding fault with the professor's "Thoughts on Chloroform." I admire the frankness and manliness of that whole communication, and never read it but with feelings of pride for the author's independence; but my trouble is in the comparison of the case of our patient, and "*the rule*" as here taught.

1st. At what stage of labor would the Prof. "benumb sensibility?"

2d. How often would he repeat?

3d. How long would he continue the anæsthetic agent?

CHESTER, Mass., 3d Oct., 1853.

